# Experiences of Domestic Violence in Adult Patients with Brain Injury: A Select Overview of Screening, Reporting, and Next Steps

**DOI:** 10.3390/brainsci14070716

**Published:** 2024-07-17

**Authors:** Jessie P. Chan, Kristen A. Harris, Arielle Berkowitz, Ally Ferber, Brian D. Greenwald, Eve M. Valera

**Affiliations:** 1 JFK Johnson Rehabilitation Institute, Edison, NJ 08820, USA; jessiep.chan@hmhn.org (J.P.C.); kristen.harris@hmhn.org (K.A.H.); arielle.berkowitz@hmhn.org (A.B.); ally.ferber@hmhn.org (A.F.); 2 Department of Physical Medicine and Rehabilitation, Rutgers Robert Wood Johnson Medical School, Piscataway, NJ 08854, USA; 3 Department of Physical Medicine and Rehabilitation, Hackensack Meridian School of Medicine, Nutley, NJ 07110, USA; 4 Massachusetts General Hospital, Charlestown, MA 02129, USA; eve_valera@hms.harvard.edu; 5 Department of Psychiatry, Harvard Medical School, Boston, MA 02115, USA

**Keywords:** traumatic brain injury, concussion, domestic violence, elder abuse, intimate partner violence, LGBTQIA+, abuse, screening, mandatory reporting

## Abstract

This select overview examines the important intersection of adult domestic violence, including intimate partner violence and elder abuse, with brain injury. Despite the high prevalence of domestic violence amongst brain injury patients, there is a notable gap in screening and management training for providers. To provide optimal patient care, brain injury medicine clinicians must screen, recognize, and treat patients who have experienced domestic violence. This select overview highlights barriers to screening, validated screening tools from other medical disciplines, and management considerations for the brain injury clinician. A suggested protocol for domestic violence screening and management, as well as recommended resources for providers and patients, is summarized.

## 1. Introduction

Intimate partner violence (IPV) and elder abuse are two forms of domestic violence that should be considered in the discussion of adult acquired brain injury [1,2,3,4,5,6]. IPV is defined as “behaviors that are intended to exert power and control over another individual, including physical, sexual, verbal, emotional, and financial abuse, and/or stalking” [7]. Elder abuse is a “single, or repeated act, or lack of appropriate action, occurring within any relationship where there is an expectation of trust which causes harm or distress to an older person” [8]. Although there are numerous forms of violence included in both IPV and elder abuse, both can lead to brain injury, including mild, moderate, or severe traumatic brain injury (TBI) and/or via strangulation [1,2,3,4,5]. Although it is known that different severities of TBI and strangulation-related brain injury are associated with domestic violence, there is insufficient research describing incidence and prevalence of specific brain injury patterns [9].

Patients who have suffered from a brain injury may present to a healthcare practitioner for evaluation of persistent symptoms, often including physiatrists, neurologists, sports medicine physicians, and neuropsychologists. It is important that brain injury medicine physicians and other healthcare practitioners feel comfortable not only recognizing the unique somatic, cognitive, and mood symptoms exhibited by patients who have experienced domestic violence but also feel capable of screening, educating, and managing this at-risk population [4,5,10,11]. Much of the literature on domestic violence screening tools originates from primary care specialties such as family medicine, internal medicine, emergency medicine, and obstetrics and gynecology [8,12,13,14]. Additionally, training for domestic violence screening is not required for brain injury medicine providers, and many may not feel comfortable with best practices for screening and next steps. This paper highlights different screening tools and considerations as an overview for clinicians evaluating patients with brain injury. In cases of positive screening, laws for reporting and national resources are outlined to address potential knowledge gaps for those treating patients with brain injury. Child abuse is another important form of domestic violence and potential cause of brain injury [15]. However, given the major distinctions between adult and pediatric populations, this overview will focus on screening tools and treatment recommendations for the adult brain injury provider. Similarly, the summarized screening tools are in the English language; however, given the importance of the published international literature, some reference is made to international studies as well.

## 2. Understanding Populations at Risk of Domestic Violence

### 2.1. Intimate Partner Violence (IPV)

IPV is a worldwide issue that impacts both men and women independent of race, socioeconomic status, religion, and ethnicity. A recent study estimated the lifetime prevalence of IPV in female patients presenting to an acquired brain injury clinic at 44%, underscoring the need for screening in this population [16]. It is important that clinicians recognize the risk factors for IPV in order to appropriately screen, educate, and provide patients with the best resources and care.

In the United States alone, there are nearly 7 million women who report experiencing IPV each year and approximately 30% of women globally experience physical or sexual IPV in their lifetime [17]. In a World Health Organization (WHO) report examining the prevalence of IPV in females over 15 years of age, lifetime exposure is reported at 29.4% in girls aged 15–19, and 37.8% in women aged 44–49 [18]. Risk factors for IPV include female gender, young age, unmarried status, ethnic minority status, low income, disability, being medically underinsured or uninsured, and a history of childhood maltreatment [19,20,21]. Patients with disabilities are at an especially high risk of IPV and can experience disability-specific forms of IPV, such as interference with, or restriction from, medical care or medications, from their caretakers and partners [22,23]. It has been estimated that women with physical disability and with mental illness are 26% and 93% more likely to experience IPV compared to their able-bodied counterparts, respectively [23]. Brain injury and IPV are closely intertwined and bidirectional risk factors [19]. In one study of women who had experienced IPV, nearly three-quarters had sustained at least one partner-related brain injury, and half reported multiple partner-related brain injuries [24]. Female brain injury survivors often report cognitive difficulties, including with decision making and impulsivity. In turn, this population may be at an increased risk for sexual, physical, and psychological abuse [9,25,26]. In addition to increased risk for brain injury, those who have experienced IPV are at an increased risk for gynecological, gastrointestinal, musculoskeletal, and cardiac complaints, as well as psychiatric disorders, including depression, anxiety, post-traumatic stress disorder (PTSD), suicidality, and substance abuse [9,20,27].

Of note, global research on domestic violence and brain injury is crucial due to variations in rates and types of violence likely secondary to cultural differences. For instance, in Andean regions of Latin America, an estimated 41% of women have experienced physical or sexual IPV [26]. Quiroz-Molinares et al. found that amongst Colombian women who had experienced IPV, 31% reported at least one brain injury and 10% sustained repetitive brain injuries over the course of an abusive relationship [26]. King et al. found that New Zealand Māori were 1.6 to 6.8 times more likely to experience IPV compared to New Zealand Europeans, Asian Indians, and Pacific Peoples [28]. Brain injury practitioners in different countries should familiarize themselves with relevant literature on IPV.

It is important to acknowledge that despite the disproportionate number of women affected, thousands of heterosexual and homosexual men also experience IPV annually and should be considered for standard screening practice [29]. Research concerning IPV, in particular IPV resulting in brain injury, among lesbian, gay, bisexual, transsexual, queer/questioning, intersex, and asexual (LGBTQIA+) individuals is notably lacking due to a multitude of factors. Recent studies have shown that the LGBTQIA+ community experiences higher rates of IPV compared to their cisgender, heterosexual counterparts [19]. In a survey conducted by Kurdyla et al., 55.2% of transgender and non-binary participants report experiencing IPV in their lifetimes [30]. These statistics are thought to be in part due to minority stressors and fear of discrimination, mistreatment, and discreditation by law enforcement, shelters, and healthcare professionals [19,31]. Of note, even with the increased focus on LGBTQIA+ communities, contemporary research primarily focuses on lesbian women and gay men, whereas bisexual and transgender individuals tend to be overlooked [31].

### 2.2. Elder Abuse

Elder abuse is an underreported and underresearched form of domestic violence. Elder abuse encompasses physical, sexual, and psychological violence, medical neglect, and financial exploitation in the setting of homes or long-term care institutions [19,29]. It is estimated that 1 in 6 people over 65 years of age experience some form of abuse [32]. Unfortunately, elderly patients receiving care within nursing homes are at increased risk of both active and passive abuse, with an estimated 64.2% of staff admitting to some form of elder abuse [33]. Although institutional abuse is a matter of great concern, over 90% of older adults in the United States live in the community, and therefore most cases of elder abuse are likely occurring outside of long-term care institutions [34].

Brain injury clinicians will be familiar with the increased prevalence of chronic subdural hematomas (SDH) in elderly patients due to age-related changes, but increased incidence alone should not preclude assessment for abuse [35]. The elderly population are also more likely to be on anticoagulation medications, increasing the chance of bruising from small accidents or disability and death from brain injury [8,35]. Risk factors for experiencing abuse include female gender, dependency on the abuser, social isolation, and history of dementia or physical disabilities. Those who experience abuse are at nearly threefold risk of hospitalization and suffer significantly higher mortality rates as compared to their peers [8]. Elder abuse is a matter of increasing concern, especially as the worldwide population of individuals older than 60 years of age rises [36].

## 3. Recognized Barriers to Screening for Domestic Violence

Most brain injury physicians and other practitioners receive little to no formal training on screening and management of different forms of domestic violence, and therefore are likely to feel uncomfortable initiating conversations with patients on this topic. In addition, patients presenting for brain injury evaluation often have a multitude of complaints, which may place time constraints on the appointment, presenting another barrier to screening. It has also been suggested that barriers to screening include lack of training, lack of privacy to screen, perceptions of IPV screening not being part of the physician role, fear of offending patients, and not knowing how to respond to a positive screen [22].

Despite the high prevalence of IPV, research suggests that only 27% of women report discussing IPV with their healthcare providers [37]. In addition, one survey of family medicine, internal medicine, and obstetrics–gynecology physicians estimated that only 10% of doctors routinely screen new patients for IPV. Likelihood increased to 79% if the patient presented to an appointment with evidence of an injury [38]. Although screening rates for IPV in brain injury clinics have not been formally studied, this has been noted as a gap in patient care given the high positive rate of identifying IPV when screening is performed [16].

One study conducted in Michigan found that while 90% of responding residency programs incorporated elder abuse education into their curricula in some way, one-third did not provide clinical training and more than half did not incorporate screening into their residency practice [39]. Another study found that more than half of surveyed physicians had no formal residency training on the topic of elder abuse detection [40]. In a study by Rhodes et al., the researchers attempted to define areas of improvement in screening by listening to audio recordings of physician–patient interactions. It was noted that oftentimes when abuse was disclosed, the healthcare worker took long pauses and quickly changed the subject, likely indicating his/her discomfort with the subject and situation [41].

Barriers to screening can vary by region and culture. A survey of healthcare providers in Nigeria found that 35% of respondents reported routinely screening for IPV and only 18.8% reported receiving training on violence. In the same study, 66% believed inquiring about IPV would be an invasion of privacy and 90% feared for their personal safety if they discussed IPV with the patient or possible perpetrator [42]. A review of the intersection of IPV and brain injury in sub-Saharan Africa found that a significant barrier to screening was the belief that IPV is a cultural problem as opposed to a medical and clinical issue that requires health-based interventions [43]. King et al. found that over the course of four years at a New Zealand hospital, less than 1% of patients who experienced IPV were assessed for brain injury, 0.6% were assessed for strangulation, and 0.5% were referred for brain injury rehabilitation services. Furthermore, under 25% of IPV reports were completed by licensed medical professionals; the majority were conducted by social workers who may not be trained in assessing signs of brain injury and strangulation [28]. In both New Zealand and Australia, an additional barrier identified was the limited hours of social workers and indigenous hospital liaison officers which presented challenges for connecting patients to culturally appropriate community resources [28,44].

## 4. Implementing Screening for Domestic Violence in Brain Injury Clinics

Identification of violent history is particularly important among patients presenting for evaluation after brain injury, including concussion, to inform management. Patients with brain injury require comprehensive care, and there may be unique considerations or referrals in persons with a history of domestic violence. Although the United States Preventive Services Task Force (USPSTF) did not find sufficient evidence to recommend screening for abuse and neglect in all older or vulnerable adults, they do recommend screening of women of reproductive age for IPV [45]. Clinicians should understand risk factors for both elder abuse and IPV, as well as available screening tools.

Prior to initiating a domestic violence screen, the clinician should have information and resources prepared in the case of a positive screen [46]. In fact, hospitals must have protocols in place to identify and assist patients experiencing abuse in order to receive accreditation [10]. The provider should have knowledge of the state mandatory reporting laws and make the patient aware of the guidelines prior to screening [22]. It is crucial to recognize who is conducting the screen, the method of the screen, who will respond to a positive screen, and how often to screen [37].

It is also essential that clinicians make themselves aware of their own biases and assumptions while screening and managing domestic violence. This is of particular importance while treating LGBTQIA+ patients; inclusive culture and language is a vital aspect of comprehensive care. It is important to acknowledge the value of both genetically related and chosen families for social, physical, and emotional connection and support. Common mistakes include assuming universal heterosexuality, monogamy, and cis-gender identification. Clinicians may also mistake intimate partners for blood relatives due to implicit biases; such presumptions can be alienating and limit open communication on topics such as domestic violence [38].

As mentioned above, the USPSTF recommends that clinicians screen women of reproductive age for IPV [45]. In addition to the USPSTF, the Women’s Preventative Services Initiative (WPSI) advises annual screening among all adolescent and adult women [47]. Screening for IPV, specifically, has been linked to an increase in intervention and subsequently better physical health. McCloskey et al. found that women who disclosed abuse were more likely to use an intervention and subsequently three times more likely to exit the abusive relationship and have improved physical health compared to women experiencing domestic violence who did not disclose the abuse to a healthcare provider [48]. According to Bonomi et al., the longer women experience IPV, the worse their health outcomes [49].

A recent study by de Souza et al. found that patients with a history of IPV-related brain injury have a higher rate of lifetime trauma exposure than those without IPV history [11]. Therefore, considerations like trauma-informed care and prioritization of symptoms that may co-occur with IPV should be taken by the treating healthcare provider. Patients may benefit from early referral to neuropsychology and psychiatry.

Despite having multiple screening options available, there is no consistent and validated tool for screening IPV in the brain injury population. Tools that can be considered for use have largely been developed from other specialties within medicine, including family medicine, emergency medicine, trauma surgery, and obstetrics and gynecology. Several papers have summarized available and validated screening tools for IPV and elder abuse, including data on sensitivity and specificity for various tools [8,12,13,14]. Given the prevalence and impact of IPV, there are several existing screening tools ranging from clinician questions during history-taking to validated measures. For elder abuse screening, there are several tools available for which psychometric properties have been evaluated but none that have been recommended by the USPSTF. An overview of existing screening tools (with ten or fewer questions) that have undergone peer-reviewed evaluation of psychometric properties is outlined in Table 1. Of note, there are other tools that are over ten questions, non-validated, or less commonly used in research that have been developed, including an untitled measure that has been recently studied in the trauma population [16].

When conducting an IPV screen, IPV-trained staff should prioritize, “(1) safety, (2) privacy, and (3) confidentiality” [61]. Clinicians should be seated at the patient’s level with the patient fully clothed and use supportive eye contact and body language [46]. IPV screening should be performed in a private setting without children or other persons present. Alternatively, if this cannot be attained, private screening can occur while escorting a patient to the restroom [62].

It is important to be aware that some patients may feel more comfortable utilizing a computer-based or written questionnaire as opposed to a face-to-face screen [20]. One review of IPV in pregnancy indicated that there was no difference in prevalence of IPV identification according to the use or not of a validated questionnaire versus patient interview, but that identification rates were higher if the purpose of measurement was related to diagnosis rather than screening [63]. It is therefore important that clinicians caring for patients with brain injury screen for IPV and ensure that patients understand the reason for screening originates not from a desire to know but from a need to provide comprehensive care for their diagnosis. There may be pragmatic considerations for implementation of standardized versus ad hoc screening in different brain injury clinic settings. While some clinics choose to have patients complete standardized questionnaires prior to their appointments, this method fails to ensure the privacy that one would want to ensure “safe” disclosure. To facilitate patient privacy, brain injury clinics could consider either in-room computer-based or written screening prior to the provider encounter.

If screening by face-to-face interview during history-taking, the language used to conduct the screen is important. It is preferable to use behaviorally specific terms such as “hit”, “slapped”, “kicked” and to avoid terms such as “battered”, “abused”, “raped”, and “domestic violence.” Avoid asking only a single question or providing only leading questions that warrant negative responses. Using the question “do you feel safe at home?” has been shown to have low sensitivity for IPV [62]. When providers use open-ended questions to invite opportunities for discussion, probe with at least one follow-up question, and act in a responsive and empathetic manner, patients are more likely to disclose abuse [41]. Even if patients have prior negative screens, it is critical to continue to have IPV screening performed at subsequent visits. Patients may be apprehensive to share IPV information due to the potential retaliation from their abuser, family, and/or community. Patients may also feel shame and embarrassment regarding the situation. The risk of disclosing this information can also cause issues regarding finances, child custody, immigration, and with the legal system [64].

Regardless of screening technique, it is recommended that clinicians and healthcare staff are trained in client centered IPV assessment and that screening is included in the electronic medical record system to prompt appropriate screening and clinical care and capture data in a standardized measure. In the case of a positive screen, the clinician should acknowledge the patient with empathy, thank them for sharing the information, and ask if they want help and/or assistance. The clinician should offer choices and resources and encourage safety planning without coercion [62]. Other recommendations include connecting patients to supportive resources regardless of disclosure. This may be difficult, however, in a brain injury clinic, where patients often receive multiple referrals to address a myriad of symptoms [65].

## 5. History and Physical Examination Considerations

### 5.1. IPV

In addition to an IPV screen, it is important for providers to use clinical judgment and recognize patient body language and non-verbal communication when the patient is accompanied by his/her caregiver/spouse. Women accompanied by a partner in the waiting room and women who receive delayed prenatal care should be recognized as patients who may be experiencing IPV [48]. A same-sex provider should be providing the screen if culturally indicated [64]. An interpreter should also be utilized in order for the screen to be conducted in the patient’s native language [62]. At the same time, one must be cognizant of the idea that a patient may feel uncomfortable disclosing information with a professional service as the interpreter may come from within the patient’s community [20].

Symptoms of brain injury and IPV can be subtle and have significant overlap, for instance headaches, confusion, memory loss, and emotional lability [19]. Patients who are experiencing IPV can have additional symptoms such as post-traumatic wound infections and genitourinary dysfunctions such as vaginal bleeding, sexually transmitted infections, pelvic pain, and urinary tract infection [19,29]. It has been suggested that addressing IPV as a chronic issue with “well-studied risk factors, natural history, and commonly associated symptoms” could be a helpful guide [20].

Amongst women who present with unverifiable injuries, the presence of head, neck, and face injuries increases the likelihood that the injuries were a result of IPV by 24% [18,66]. In South Korea, a primary care center found that 79.9% of patients who reported experiencing domestic violence presented with physical injuries, the majority of which were of the face, upper extremities, and head and neck [67]. Strangulation, a life-threatening act commonly occurring in IPV, leaves no visible marks on the neck in approximately half of cases but may present with signs like petechial hemorrhage, throat pain, vocal hoarseness, painful swallowing, and mental status changes due to hypoxia [68,69]. In New Zealand, King et al. found that nearly half of people who experienced IPV reported being choked, and over a third were “knocked out” [28]. While computed tomography (CT) and magnetic resonance imaging (MRI) are less effective in identifying mild brain injuries, they can be useful in the detection of maxillofacial injuries and significant brain contusions or bleeds [29].

### 5.2. Elder Abuse

In the case of elder abuse, the clinician should look for signs of anxiety and poor eye contact. If lab testing is performed, signs of dehydration, low medication levels, and evidence of misused harmful or sedating prescription drugs or illicit substances, such as in the urine, blood, or hair may necessitate further investigation into elder abuse [70]. Symptoms of physical abuse in the elderly population can be difficult to distinguish from accidental injury from falls or side effects from medications [71]. Red-flag symptoms for elder abuse would include multiple fractures at various stages of healing or several acute or acute-on-chronic subdural hematomas in different locations, occurring at different times, or associated with a skull fracture [35]. Some physical symptoms that should raise suspicion for mistreatment include welts, bite marks, long bone or facial fractures, posterior rib fractures, dehydration, extensive decubitus ulcers, sexually transmitted infections, and/or poor hygiene [8,35].

## 6. Reporting Domestic Violence

The importance of physician and healthcare practitioner education on identifying signs and symptoms of domestic violence, as well as an approach to discussing and reporting the topic, cannot be overstated. Mandatory laws for reporting are drastically different depending on type of abuse and the location of medical practice. When a patient presents with certain types of injuries, physicians are mandated to report the case in most states. At least 40 states require reporting when the injury appears to be a result of deadly weapons, whereas at least 18 states require reporting if the injury is a result of an illegal act [72]. However, patients may not fulfill the criteria for mandatory IPV reporting when presenting to physicians for nonspecific chronic symptoms that could be a result of a brain injury from IPV. This could include, but is not limited to, headaches, difficulty concentrating, poor sleep, or chronic pain [72].

Ultimately, mandated reporting of IPV is ethically challenging as patients may suffer as a result. Multiple organizations, including the American College of Obstetricians and Gynecologists (ACOG), American Medical Association (AMA), American College of Emergency Physicians, and American Academy of Pediatrics (AAP) are opposed to mandated reporting of IPV [73]. For example, in New Jersey, the authors’ state of practice, stat 2C:58-8 requires physicians to report any case of injury caused by firearm, destructive device, explosive, or weapon or any wound or burn immediately to police authorities in the municipality where the patient reporting is located. However, there are no official reporting protocols for IPV in New Jersey [72]. Very few states have established protocols or screening requirements for healthcare providers or facilities in cases of suspected IPV.

Though not in the majority, some states have initiative in place that could potentially be implemented elsewhere. For example, the Ohio Department of Health and Domestic Violence Network are working on training programs in addition to placing small safety planning cards in workplace restrooms. Training in Massachusetts through the Domestic Violence Screening Care, Referral, and Information Project aims to improve the quality of care for women and children affected by domestic violence identified through screening, protocol development, and referrals to resources. West Virginia also has enacted statewide training for behavioral health and substance abuse providers for domestic violence. New Jersey does not have protocols or screening instated. New Jersey stat 52:27D-43.36 holds the director of the Division on Women in the Department of Community Affairs and the Health and Senior Services accountable for promoting public awareness of IPV through campaigns among the general public and healthcare community [73]. These campaigns are intended to also assist those who have experienced IPV with resource connectivity.

**Figure 1:**
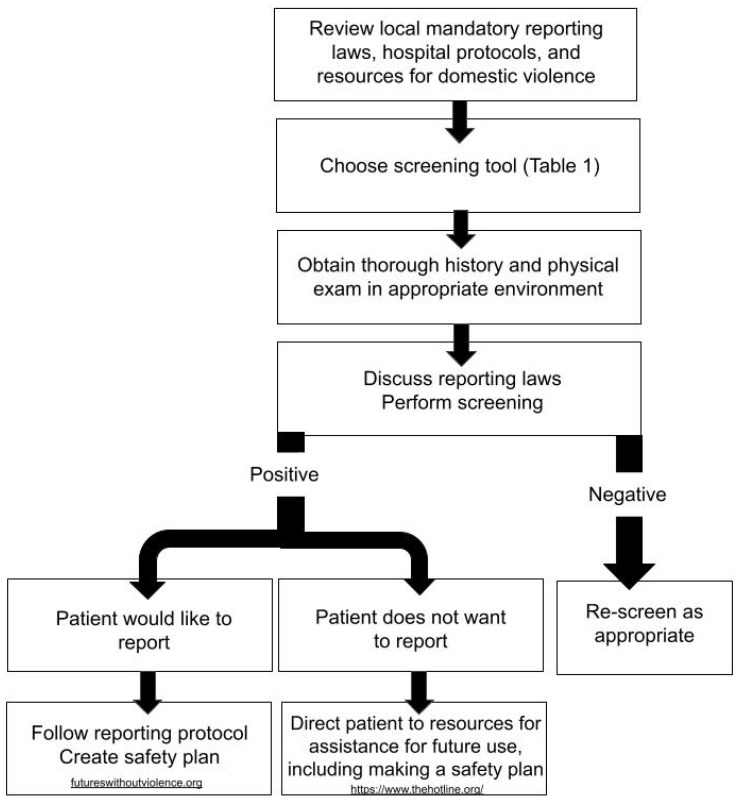
Flowchart for women of reproductive age or suspected domestic violence.

Healthcare providers and medical centers should have knowledge of local mandatory reporting laws and guidelines in the case of a positive IPV screen [46]. Further information on state specific reporting laws, protocols, screening, and training can be found through Futures Without Violence and other resources [73]. Patient readiness to report should be assessed and guidance from the provider should be provided accordingly (Figure 1).

Futures Without Violence and individual state resources (https://www.futureswithoutviolence.org/wp-content/uploads/Compendium-4th-Edition-2019-Final.pdf, accessed on 7 April 2024) are presented here.

Despite lack of evidence supporting the efficacy of reporting adult abuse, there are federal and state statutes in all states to protect adults from elder abuse, neglect, or financial exploitation through adult protective services [74,75]. Reports can be made to law enforcement for imminent danger, adult protective services, or state-specific licensing agencies [76]. All states except New York have distinguished specific mandated reporters; however, these lists vary by state. Fifteen states have universal reporting, meaning all people are required to report elder abuse if suspected; physicians are considered required reporters in nearly all states. The definition of adult abuse is broader compared to child abuse, which leads to inconsistency in opinions on if reporting should be mandated and who should be a mandated reporter for adult abuse. There is also controversy regarding the risk of causing more harm than good by reporting, such as stigmatization or increased tension and conflict between the parties involved [77].

The aging population’s decline in health also poses problems in mandating reporting as certain health conditions can appear as a form of elder abuse or neglect, such as bruising from coagulation disorders, fractures from osteoporosis, or weight loss from cancer. Filing a false report in situations such as those creates fear among potential reporters. There is agreement amongst advocates that training on identification and reporting procedures would be beneficial; however, the type of training that will be most effective has not been established [75].

Mandatory Reporting Requirements for Elderly and/or Vulnerable Persons (https://www.napsa-now.org/wp-content/uploads/2016/05/Mandatory-Reporting-Chart-Updated-December-2015-FINAL.pdf, accessed on 15 April 2024) are presented here.

## 7. Resources for Affected Individuals

A safety plan is an individualized practical plan established in preparation for an individual to leave an abusive situation. This includes steps on how to leave, what to do after leaving, how to make friends and family aware, how to cope with the situation, and how to access resources. Creating this safety plan can look different given people’s unique situations, such as presence of children, pets, pregnancy, or whether or not the involved parties live together. The National Domestic Violence Hotline is a valuable resource in the United States that can be reached by calling 800-799-7233, texting START to 88788, or live chat online. Online resources are available for creating a safety plan, including child and pet safety, local shelter information, emergency shelter transportation, financial support, childcare assistance, counseling, support groups, food and health services, legal assistance, sexual assault services, and more [78].

In addition to physically preparing for safety, emotional safety is also necessary. Portions of the safety plan should include law enforcement and protective orders and legal resources if appropriate. Lastly, the National Domestic Violence Hotline contains various resources for different populations, including the deaf community, Native American community, Black community, Latinx community, Asian American and Pacific Islander communities, LGBTQIA+ youth community, Jewish community, and more. State-specific information can also be found in this central resource [78].

The National Domestic Violence Hotline (https://www.thehotline.org/, accessed on 10 April 2024) resource is presented here.

## 8. Conclusions

Domestic violence, including IPV and elder abuse, is an important and under-recognized cause of brain injury. Domestic violence screening rates have not been formally studied in brain injury clinics; however, data on screening rates from other medical specialties suggest that current screening rates are low. Given the prevalence of IPV-related brain injury and unique presentation and management considerations, brain injury clinicians should familiarize themselves with risk factors for violence, available screening tools, best practices, and management considerations.

## 9. Future Directions

Despite the prevalence of domestic violence, there is still much work to be performed in improving screening tools in patients with brain injury. Specific areas for further research are highlighted below:

### 9.1. Screening

Existing screening tools developed from other specialties have not been specifically validated in the brain injury population. Future studies could evaluate the use of common domestic violence screening tools in the brain injury population. Recently, efforts have been made to improve brain injury screening tools for assessment of IPV-related brain injury. The Brain Injury Screening Questionnaire IPV (BISQ-IPV) has been introduced to screen for IPV-related brain injury, and the HELPS brain injury screening tool has been modified to include strangulation items for IPV. Of note, both of these updates are to existing brain injury screening tools, which may limit their applicability in a brain injury clinic setting where patients are presenting for evaluation of an already-known diagnosis [79,80].

Further research is warranted to better understand current screening and management practices for domestic violence in brain injury clinics. Although current screening rates and barriers to screening for domestic violence have been studied in other medical specialties, there is a tremendous opportunity to better understand these factors in patients presenting for evaluation of brain injury. Comparative studies between validated computer-based or paper-based screening tools versus face-to-face ad hoc screening could be undertaken in the brain injury population. Additionally, further research is warranted to understand incidence of different severities and forms, namely traumatic and strangulation-related brain injury associated with domestic violence.

### 9.2. Training

The literature suggests that lack of clinician training is a major barrier to screening for domestic violence. As such, training for domestic violence-related brain injury should be standardized across physician and healthcare provider training programs. Future studies could evaluate the efficacy of educational programs on domestic violence programs for clinicians, including impact on screening rates, and clinician-reported comfort with screening and identification of domestic violence. Furthermore, given the importance of prepared resources in case of a positive screen, future research could evaluate the impact of educational programs for clinic staff.

### 9.3. Reporting

Future studies could evaluate brain injury clinician familiarity with domestic violence reporting laws, rates of domestic violence reporting in brain injury patients, as well as impact on patient care.

### 9.4. Gender and Culturally Sensitive Care

The existing literature indicates that the rates and patterns of domestic violence vary globally and culturally. Future research could examine domestic violence and brain injuries within different communities and current beliefs, clinical practices, and community resources. Studies could build upon that knowledge to investigate and develop culturally- and resource-sensitive practices in order to effectively screen and support patients experiencing domestic violence and guide healthcare providers in providing optimal care.

Much of the current research on injury-related IPV focuses on women who have experienced IPV at the hands of men. Future studies could investigate patterns of injury and behavior in men who experience IPV from female partners, as well as the impact of stigmatization on screening and reporting.

Research on IPV and brain injuries involving LGBTQIA+ persons remains limited [81]. There is a need for increased focus on this topic, especially in regards to bisexual, nonbinary, and transgender individuals. Future studies could additionally expand upon the intersectionality of domestic violence, brain injuries, marginalization, and health inequities within the community.

## Figures and Tables

**Table 1:** Screening tools for domestic violence.

Tool	Components	Originating Discipline	Notes
Abuse Assessment Screen (AAS) [50] Ongoing Abuse Screen (OAS)/Ongoing Violence Assessment Tool (OVAT) [51]	5 questions 4 questions	Obstetrics and Gynecology	
Addressing Reproductive Coercion in Health Settings (ARCHES) [52]	6 questions	Obstetrics and Gynecology	
Humiliation, Afraid, Rape and Kick (HARK) [53]	4 questions	General Practice	Recommended by USPSTF
Hurt Insulted Threatened or Screamed at Instrument (HITS) [54] E-HITS [55]	4 questions 5 questions	Family Medicine	Recommended by USPSTF Validated Spanish language version available
Partner Violence Screen [56]	3 questions	Emergency Medicine	Recommended by USPSTF
Relationship Assessment Tool (RAT)/Women’s Experience with Battering (WEB) [57]	10 questions	Public Health	Validated Spanish language version available
SAFE-T [58]	5 questions	Emergency Medicine	Indirect questions only, more effective at ruling out cases of IPV
Women Abuse Screening Tool (WAST) [59] WAST-SF [59]	8 questions 2 questions	Family Medicine	Recommended by USPSTF Validated Spanish language version available
Elder Abuse Suspicion Index (EASI) [60]	6 questions	Family Medicine	

Caption: A brief overview of available domestic violence (including IPV and elder abuse) screening tools that can be considered for use in the clinical setting.

## Data Availability

No new data were created or analyzed in this study. Data sharing is not applicable to this article.
